# Instrumented Assessment of Motor Performance Fatigability During the 6-Min Walk Test in Mildly Affected People With Multiple Sclerosis

**DOI:** 10.3389/fneur.2022.802516

**Published:** 2022-05-09

**Authors:** Kim-Charline Broscheid, Martin Behrens, Patrizia Bilgin-Egner, Anita Peters, Christian Dettmers, Michael Jöbges, Lutz Schega

**Affiliations:** ^1^Health and Physical Activity, Department of Sport Science, Institute III, Otto von Guericke University Magdeburg, Magdeburg, Germany; ^2^Department of Orthopedics, University Medicine Rostock, Rostock, Germany; ^3^Kliniken Schmieder Konstanz, Konstanz, Germany

**Keywords:** MS, fatigue, attractor method, minimum toe clearance, gait kinematics

## Abstract

There are conflicting results regarding the changes in spatio-temporal gait parameters during the 6-min walk test (6MWT) as indicators of gait-related motor performance fatigability (PF) in people with Multiple Sclerosis (pwMS). To further analyze if gait-related motor PF can be quantified using instrumented gait analysis during the 6MWT, we investigated: (i) whether gait parameters recorded during the first or second minute were more stable and thus the better baseline to assess motor PF and (ii) if the minimum toe clearance (MTC) together with “classical” spatio-temporal gait parameters can be used to quantify motor PF in pwMS. Nineteen mildly affected pwMS [12 women/7 men; 47.8 ± 9.0 years; the Expanded Disability Status Scale (EDSS): 2.7 ± 1.0] and 24 healthy controls (HC; 15 women/9 men; 48.8 ± 7.6 years) completed the 6MWT equipped with inertial measurement units. Data were analyzed using the attractor method to compare the stability of gait parameters and, besides “classical” spatio-temporal gait parameters, the MTC was calculated as a potential new marker for motor PF in pwMS as this was shown in healthy older adults. It was found that (i) gait parameters were more stable in the second than in the first minute and (ii) gait-related motor PF could not be detected based on spatio-temporal gait parameters, including the MTC. Descriptive analysis indicated a decrease in MTC variability, which is assumed to be indicative for motor PF, toward the end of the 6MWT in some pwMS. Future studies should investigate gait parameters for the assessment of motor PF in pwMS recorded during more intense and/or longer walking protocols, taking the level of disability into account. Furthermore, using gait parameters recorded in the first minute of the 6MWT as a baseline for the assessment of motor PF should be avoided.

## Introduction

Multiple Sclerosis (MS) is an autoimmune inflammatory neurodegenerative disease with diverse symptoms that depend on the lesion site. The disease is often accompanied by motor deficits (1) and fatigue (2) that limit locomotion and quality of life. Over 75% people suffer from fatigue and 40% of people with MS (pwMS) report that this is the most limiting symptom (2). Based on the definition of Kluger et al. (3) and Enoka and Duchateau (4), fatigue can be assessed either as a trait or a state characteristic. While trait fatigue describes the fatigue perceived by an individual over a longer period of time, state fatigue refers to the acute and temporary change in motor and/or cognitive performance (performance fatigability/PF) and various perceptions that emerge during a defined sustained motor and/or cognitive task (perceived fatigability).

The extent of motor PF induced by motor tasks is determined by changes in the muscle activation characteristics and the contractile function of the involved muscles. Perceived fatigability during motor tasks depends on the psychological status of an individual and the homeostatic perturbations induced by the motor task (4).

There are a variety of methods to quantify motor PF in pwMS but currently, no gold standard exists. Several exercise models were used to assess motor PF in pwMS, which were recently summarized by Severijns et al. (5) and van Geel et al. (6). They have shown that single-joint exercises and physical activities, such as walking, which are close to activities of daily life, were used to induce motor PF. For the latter approach, the 6-min walk test (6MWT) is frequently used (5, 6). However, studies using this paradigm reported discrepant results regarding the discriminative value for the assessment of motor PF in pwMS. In this regard, some studies have focused on the walking velocity (e.g., distance walked index/DWI (7) or deceleration index) (8). A recently published study by Shema-Shiratzky et al. demonstrated that walking velocity did not change significantly across the 6MWT and is thus of limited relevance as a standalone marker for the quantification of motor PF in pwMS. Moreover, they suggested that other kinematic parameters, such as cadence, stride time variability, and gait complexity (sample entropy of the 3D acceleration and gyroscope data), might be more appropriate for this purpose (9).

Besides these variables, a promising spatial gait parameter to quantify gait-related motor PF has not yet been investigated during the 6MWT in pwMS, i.e., the minimum toe clearance (MTC) and its variability. The MTC describes the minimum vertical toe to ground distance in the mid-swing phase (10) and is related to the risk of falling (11). If it approaches zero, the probability of tripping is very high. The MTC variability is able to differentiate between different populations, e.g., young and elderly and fallers and non-fallers (11, 12). A study by Nagano et al. has demonstrated that the MTC variability becomes smaller during prolonged walking in contrast to the variability of step width in older adults. Therefore, it was assumed that the MTC seems to be prioritized with increasing motor PF to reduce the risk of falling (13). Since the hip flexors are weaker (14) and the toe height is increased during treadmill walking in pwMS when compared to healthy individuals (15), it is conceivable that the MTC is sensitive to motor PF in pwMS as shown for healthy older adults (13).

However, the existing approaches have mostly used the first minute of walking (except the deceleration index) as a baseline to quantify gait-related motor PF. This might not be favorable, since people start from a standing position and gait initiation has a high impact on gait measures during the initial meters walked (16). Furthermore, it is known that dynamic cyclic systems, such as running and walking, need a certain time to become stable (transient effect) (17). To evaluate the gait stability, the attractor method introduced by Vieten et al. can be applied (18). According to Newell et al. “Attractors represent equilibrium regions in the geometric space (called state space) that are formed by the relevant variables describing the movement dynamic […].” (19). The stability of cyclic movements, such as walking, can be described by limit-cycle attractors (18), which are “[…] a regular oscillation to which all trajectories converge […]” (19).

In summary, gait parameters for quantifying motor PF during walking in pwMS are controversially discussed and there is no agreement about the most indicative parameter or combination of parameters (6). Moreover, it is not clear whether the second minute is more appropriate as the reference baseline for quantifying gait-related motor PF than the first minute of the 6MWT.

Therefore, the aim of this study was to investigate (i) the gait stability during the first 2 min of the 6MWT using the attractor method and (ii) if the MTC and its variability together with classical spatio-temporal gait parameters can be used to quantify gait-related motor PF over the course of the 6MWT in mildly affected pwMS. We expected that gait parameters are more stable in the second minute than in the first one. Furthermore, we assumed that spatio-temporal gait parameters deteriorate over the course of the 6MWT and that the MTC is prioritized (decreased variability), indicating motor PF in mildly affected pwMS.

## Materials and Methods

### Participants

For this cross-sectional study, 19 pwMS and 25 healthy controls (HC) with similar age and sex were included. All pwMS had a confirmed MS diagnosis according to the revised McDonald criteria (20). For inclusion in the study, subjects should be able to walk 300 m without a walking aid and the Expanded Disability Status Scale (EDSS) (21) should not be > 4.5. Furthermore, the last acute episode and the last dose of cortisone should be taken at least 1 month ago. The exclusion criteria for the HC and pwMS were orthopedic, cardiovascular, and neurological diseases with the exception of MS. The Ethics Committee of the Medical Faculty of the Otto von Guericke University (OvGU) Magdeburg (Germany) approved the study (no.: 116/18).

### Study Procedure

The study was conducted at the Kliniken Schmieder Konstanz (Germany) in cooperation with the OvGU Magdeburg (Germany). The pwMS were recruited by health professionals at the beginning of their rehabilitation. The HC were recruited from local citizens. In a first interview, the participants were informed about the study, and written informed consent was obtained. To assess the perceived MS-induced walking disability, the pwMS filled out the German version of the 12-Item Multiple Sclerosis Walking Scale (MSWS-12) (22). Trait fatigue was documented with the Fatigue Scale for Motor and Cognitive function (FSMC) (23). Gait analysis was performed using two inertial measurement units (sampling frequency 120 Hz) (MTw, Xsens Technologies B.V., Netherlands) placed dorsally at each foot (24). For the attractor-based gait analysis, continuous walking was needed so that the 6MWT was performed on a circular oval quite corridor at the clinic with a fixed circumference of 34 m. The subjects should walk as fast as possible but safely and were accompanied by a physiotherapist. No walking aid was used. Every minute was announced loudly by the test instructor. Ratings of perceived exhaustion (RPE) on a Borg scale (25) (6: no exhaustion, 20: maximal exhaustion) were recorded before and after the 6MWT to quantify perceived fatigability.

### Gait Data and Processing

To determine which minute of the 6MWT is more stable, the non-linear limit-cycle attractors were calculated utilizing the 3D acceleration and rotation data of the feet for each minute. The outcome parameters were the relative difference between two limit-cycle attractors [δM (1/s)], the relative difference between the variability of two limit-cycle attractors [δD (m/s^2^)], and the absolute variability [D (m/s^2^)] of each minute. In this study, the second minute was compared with the other minutes of the 6MWT: δM/δD_2vs1min_, δM/δD_2vs3min_, δM/δD_2vs4min_, δM/δD_2vs5min_, and δM/δD_2vs6min_. The equations are described in the study by Vieten et al. (18).

To assess motor PF over the 6MWT, the following spatio-temporal gait parameters were calculated for each minute: stride length, stride, stance and swing time, gait velocity, the MTC, and the respective variability [coefficient of variation/CV (%): standard deviation (SD)/mean × 100]. Gait parameters were calculated according to the algorithm of Hamacher et al. (24) based on 3D rotation and acceleration data of the feet. The first 2.5 m of the 6MWT were not considered to reduce the impact of gait initiation. Derived from the gait velocity, the walking distance per minute was constructed to calculate the DWI [decline in walk distance from the first (here also second) to the last minute of the 6MWT in percent]. A decline of more than 10% is interpreted as an indicator of motor PF (26). All calculations were done in MATLAB (The Mathworks^Ⓡ^, Version R2019b, Natick, USA).

### Statistical Analysis

The statistical analysis was performed with the IBM SPSS software (Version 26, Chicago, USA). Normal distribution was checked with the Shapiro-Wilk test. Despite partially non-normally distributed data, repeated measures ANOVAs with the factors time (each minute of the 6MWT for the gait parameters and pre and post for RPE) and groups (pwMS and HC) were conducted. According to Blanca et al., the ANOVA is robust against violation of normal distribution (27). The effect size for partial eta-squared η_p_^2^ was determined (small > 0.01, medium > 0.06, and large > 0.14 effect) (28). Bonferroni *post-hoc* tests were performed if significant main or interaction effects were found. The effect size Cohen's d was calculated for the within-group comparisons (small > 0.2, medium > 0.5, and large > 0.8 effect size) (28, 29). The bias-corrected Hedge's g was chosen for the between-groups comparisons (small > 0.2, medium > 0.5, and large > 0.8 effect size) (29). The level of significance was set at *p* ≤ 0.05. A trend was interpreted with *p* ≤ 0.1. For all repeated measures ANOVAs, the Greenhouse-Geisser correction was applied since the assumption of sphericity was not given.

## Results

### Descriptive Data and Clinical Outcome Measures

Data of 19 pwMS (12 women/7 men; 47.8 ± 9.0 years) could be analyzed (Table 1). The pwMS included were mildly affected (EDSS of 2.7 ± 1.0) and suffered from MS for 13.8 ± 8.6 years since the first diagnosis. Fifteen pwMS exhibited the relapsing-remitting, two primary and two secondary progressive MS types. The HC group consisted of 24 participants (15 women/9 men; 48.8 ± 7.6 years). One participant had to be excluded because of missing data.

**Table 1:** Descriptive subject data and clinical measures.

	**pwMS (*N* = 19)**	**HC (*N* = 24)**
Age (years)	47.8 ± 9.0	48.8 ± 7.6
Sex (f/m)	12/7	15/9
Height (cm)	173.6 ± 9.3	172.7 ± 8.4
Weight (kg)	75.7 ± 11.1	73.9 ± 13.0
Expanded Disability Status Scale	2.7 ± 1.0	n.a.
MS-type (RR/PP/SP)	15/2/2	n.a.
Disease duration (years)	13.8 ± 8.6	n.a.
6MWT (m)	478.1 ± 60.7	641.4 ± 56.5
DWI_1−6_ ( ≤ - 10%/−10-0%/≥ 0%)	4/10/5	0/15/9
DWI_2−6_ ( ≤ - 10%/−10-0%/≥ 0%)	1/9/9	0/14/10
MSWS-12 (%)	54.7 ± 23.2	n.a.
FSMC-total	67.4 ± 18.2	n.a.
Physical subscale	34.0 ± 9.1	n.a.
Cognitive subscale	33.4 ± 10.3	n.a.
RPE pre	10.5 ± 3.3	8.7 ± 1.8
RPE post	12.3 ± 3.1	9.9 ± 2.5

*pwMS, people with Multiple Sclerosis; HC, healthy controls; f, female; m, male; RR, relapsing remitting; PP, primary progressive; SP, secondary progressive; 6MWT, 6-min walk test; DWI_1-6_, distance walked index from min 1 to 6; DWI_2-6_, distance walked index from min 2 to 6; MSWS-12, 12-Item Multiple Sclerosis Walking Scale; FSMC, Fatigue Scale for Motor and Cognitive function; RPE, rating of perceived exhaustion; n.a., not applicable*.

The pwMS reported moderate perceived walking limitations [12-Item MSWS: 54.7 ± 23.2%]. Three pwMS declared that they had no walking restrictions. The FSMC revealed that the pwMS included suffered severely from cognitive as well as physical perceived trait fatigue with an overall score of 67.4 ± 18.2 (scale 20–100; ≥ 43 mild/≥ 53 moderate/≥ 63 severe fatigue). Thirteen pwMS rated their motor fatigue as severe, three as moderate, and only one as mild.

### Gait Stability – Attractor Method

For all three parameters, δM, δD, and D, a significant time effect (η_p_^2^ = 0.15, *F*_1.215, 49.832_ = 7.483, *p* = 0.006/η_p_^2^ = 0.10, *F*_2.554, 104.713_ = 4.517, *p* = 0.008/η_p_^2^ = 0.13, *F*_2.693, 110.394_ = 6.326, *p* = 0.001) was found (Table 2). Furthermore, a significant group effect could be demonstrated for δM (η_p_^2^ = 0.19, *F*_1.000, 41.000_ = 9.819, *p* = 0.003).

**Table 2:** Attractor-based gait parameters (mean ± SD) for each minute of the 6-min walk test and repeated measures ANOVAs (*p*-values and partial eta^2^ effect size).

		**Performance per minute**						***p*** **-values**			**Partial eta^2^**		
**Gait parameter**	**Group**	**Min 1**	**Min 2**	**Min 3**	**Min 4**	**Min 5**	**Min 6**	**Time**	**Group**	**Time × group**	**Time**	**Group**	**Time × group**
D (m/s^2^)	pwMS	3.40 ± 1.19	3.05 ± 0.81	3.05 ± 0.87	3.19 ± 1.09	3.21 ± 1.14	3.24 ± 1.05	**0.001**	0.198	0.577	**0.13**	0.40	0.02
	HC	3.02 ± 0.78	2.78 ± 0.72	2.82 ± 0.74	2.80 ± 0.71	2.86 ± 0.69	2.85 ± 0.69						
		**Min 2 vs. 1**	**Min 2 vs. 3**	**Min 2 vs. 4**	**Min 2 vs. 5**	**Min 2 vs. 6**		
delM (1/s)	pwMS	2.33 ± 1.33	1.12 ± 0.54	1.49 ± 0.72	2.10 ± 2.37	2.49 ± 3.11		**0.006**	**0.003**	0.174	**0.15**	**0.193**	0.04
	HC	1.03 ± 0.38	0.67 ± 0.22	0.84 ± 0.36	1.00 ± 0.50	1.18 ± 0.62							
delD (m/s^2^)	pwMS	0.90 ± 1.52	0.05 ± 0.95	0.38 ± 1.22	0.75 ± 1.72	0.78 ± 2.03		**0.008**	0.230	0.376	**0.10**	0.035	0.02
	HC	0.68 ± 0.64	0.11 ± 0.73	0.01 ± 0.71	0.30 ± 0.77	0.13 ± 0.83							

*pwMS, people with Multiple Sclerosis; HC, healthy controls; SD, standard deviation; delM, difference between two limit-cycle attractors; delD, differences between the variability of two limit-cycle attractors; bold, p ≤ 0.1*.

Bonferroni *post-hoc* within-group comparisons showed a significant difference between δM_2vs3min_ and δM_2vs4min_ in both groups and between δM_2vs3min_ as a reference and δM_2vs1min_, δM_2vs5min_, and δM_2vs6min_, respectively (*p* < 0.05, d = 0.5–1.2) in pwMS (Table 3). Moreover, a significant difference was demonstrated between δD_2vs3min_ and δD_2vs1min_ (*p* = 0.009, d = 0.7) in pwMS and δD_2vs3min_ and δD_2vs4min_ in HC (*p* = 0.021, d = 0.8). The groups significantly differed in δM_2vs1min_, δM_2vs3min_, δM_2vs4min_, and δM_2vs5min_ with medium to large effect sizes (*p* < 0.05, g = 0.7–1.4; Table 4).

**Table 3:** *Post-hoc* within-group comparisons of the second minute with the other minutes of the 6-min walk test and of the difference between the limit-cycle attractors (delM_2vs3_) and their variability (delD_2vs3_) of min 2 and 3 with the differences of the other minutes of the 6-min walk test (*p* and Cohen's d effect size) only for the significant repeated measures ANOVAs.

**Gait parameter**	**Group**	**Min 1**		**Min 3**		**Min 4**		**Min 5**		**Min 6**	
		***p***	**d**	***p***	**d**	***p***	**d**	***p***	**d**	***p***	**d**
MTC	pwMS	1.000	0.4	1.000	0.2	1.000	0.2	1.000	0.2	1.000	0.2
Stance time		1.000	0.2	1.000	0.1	1.000	0.2	1.000	0.1	1.000	0.2
Stride time		1.000	0.2	1.000	0.1	0.716	0.3	1.000	0.2	1.000	0.3
Velocity		1.000	0.1	1.000	0.1	1.000	0.1	1.000	0.1	1.000	0.1
Velocity_CV_		1.000	0.2	1.000	0.1	1.000	0.2	1.000	0.1	1.000	0.1
D		0.115	0.5	1.000	0.0	1.000	0.2	0.996	0.3	0.328	0.4
MTC	HC	1.000	0.3	**0.003**	**0.8**	**0.001**	**1.0**	**0.001**	**1.1**	0.406	1.4
Stance time		**0.016**	**0.7**	1.000	0.2	1.000	0.2	1.000	0.1	1.000	0.0
Stride time		0.137	0.6	1.000	0.2	1.000	0.2	1.000	0.1	1.000	0.0
Velocity		0.776	0.3	1.000	0.3	1.000	0.3	1.000	0.2	1.000	0.1
Velocity_CV_		0.114	0.5	1.000	0.2	1.000	0.1	1.000	0.0	1.000	0.2
D		0.545	0.9	1.000	0.2	1.000	0.1	1.000	0.4	1.000	0.3
		**Min 2 vs. 1**		**Min 2 vs. 4**		**Min 2 vs. 5**		**Min 2 vs. 6**		
delM_2vs3min_	pwMS	**<0.001**	**1.2**	**<0.001**	**1.2**	**0.044**	**0.5**	**0.036**	**0.5**	
delD_2vs3min_		**0.009**	**0.7**	1.000	0.3	0.102	0.5	0.320	0.4	
delM_2vs3min_	HC	0.122	1.2	**0.021**	0.8	1.000	0.9	1.000	1.1	
delD_2vs3min_		**0.098**	**0.7**	1.000	0.1	1.000	0.2	1.000	0.0	

*p, p-value; d, Cohen's d; MTC, minimum toe clearance; HC, healthy controls; pwMS, people with Multiple Sclerosis; CV, coefficient of variation; delM, difference between two limit-cycle attractors; delD, differences between the variability of two limit-cycle attractors; D, absolute variability around one limit-cycle attractor; bold, p ≤ 0.05*.

**Table 4:** *Post-hoc* between-group comparisons for each minute of the 6-min walk test (*p* and Hedge's g effect size).

**Gait parameter**	**Min 1**		**Min 2**		**Min 3**		**Min 4**		**Min 5**		**Min 6**	
	***p***	**g**	***p***	**g**	***p***	**g**	***p***	**g**	***p***	**g**	***p***	**g**
MTC	**0.025**	**0.7**	**0.004**	**0.9**	**0.017**	**0.8**	**0.018**	**0.7**	**0.025**	**0.7**	0.984	0.0
MTC_CV_	0.078	**0.5**	**0.011**	**0.8**	**0.004**	**0.9**	**0.034**	**0.7**	**0.014**	**0.8**	0.178	0.4
Stride length	**<0.001**	**1.8**	**<0.001**	**1.7**	**<0.001**	**1.8**	**<0.001**	**1.9**	**<0.001**	**1.9**	**<0.001**	**2.0**
Stride length_CV_	0.403	0.3	**<0.001**	**1.3**	**0.005**	**0.9**	**0.001**	**1.0**	**0.009**	**0.8**	**0.003**	**1.0**
Stance length	**<0.001**	**2.1**	**<0.001**	**2.1**	**<0.001**	**2.0**	**<0.001**	**1.9**	**<0.001**	**1.6**	**<0.001**	**1.7**
Stance length_CV_	0.064	**0.6**	**0.007**	**0.8**	**0.01**	**0.8**	**0.001**	**1.0**	**0.004**	**0.9**	**0.005**	**0.9**
Swing length	**0.005**	**0.9**	**0.004**	**0.9**	**0.005**	**0.9**	**0.002**	**1.0**	**0.01**	**0.8**	**0.011**	**0.8**
Swing length_CV_	**0.035**	**0.6**	**0.02**	**0.7**	0.069	**0.6**	**0.011**	**0.8**	**0.022**	**0.7**	**0.04**	**0.6**
Stride time	**<0.001**	**1.8**	**<0.001**	**1.7**	**<0.001**	**1.6**	**<0.001**	**1.6**	**<0.001**	**1.3**	**<0.001**	**1.3**
Stride time_CV_	0.35	0.3	**0.013**	**0.8**	0.073	**0.5**	**0.009**	**0.8**	**0.017**	**0.7**	**0.048**	**0.6**
Velocity	**<0.001**	**2.6**	**<0.001**	**2.4**	**<0.001**	**2.4**	**<0.001**	**2.5**	**<0.001**	**2.3**	**<0.001**	**2.3**
Velocity_CV_	0.361	0.3	**<0.001**	**1.3**	**0.006**	**0.9**	**<0.001**	**1.2**	**0.003**	**0.9**	**0.002**	**1.0**
D	0.211	0.4	0.250	0.3	0.347	0.3	0.160	0.4	0.227	0.4	0.152	0.4
	**Min 2 vs. 1**		**Min 2 vs. 3**		**Min 2 vs. 4**		**Min 2 vs. 5**		**Min 2 vs. 6**		
delM	**<0.001**	**1.4**	**<0.001**	**1.1**	**<0.001**	**1.2**	**0.032**	**0.7**	**0.051**	**0.6**	
delD	0.531	0.2	0.805	0.1	0.223	0.4	0.258	0.3	0.161	0.4	

*p, p-value; g, Hedge's g; MTC, minimum toe clearance; HC, healthy controls; pwMS, people with Multiple Sclerosis; CV, coefficient of variation; delM, difference between the limit-cycle attractors of 2 min; delD, differences between the variability of two limit-cycle attractors; D, absolute variability; bold, p ≤ 0.05*.

**Figure 1:**
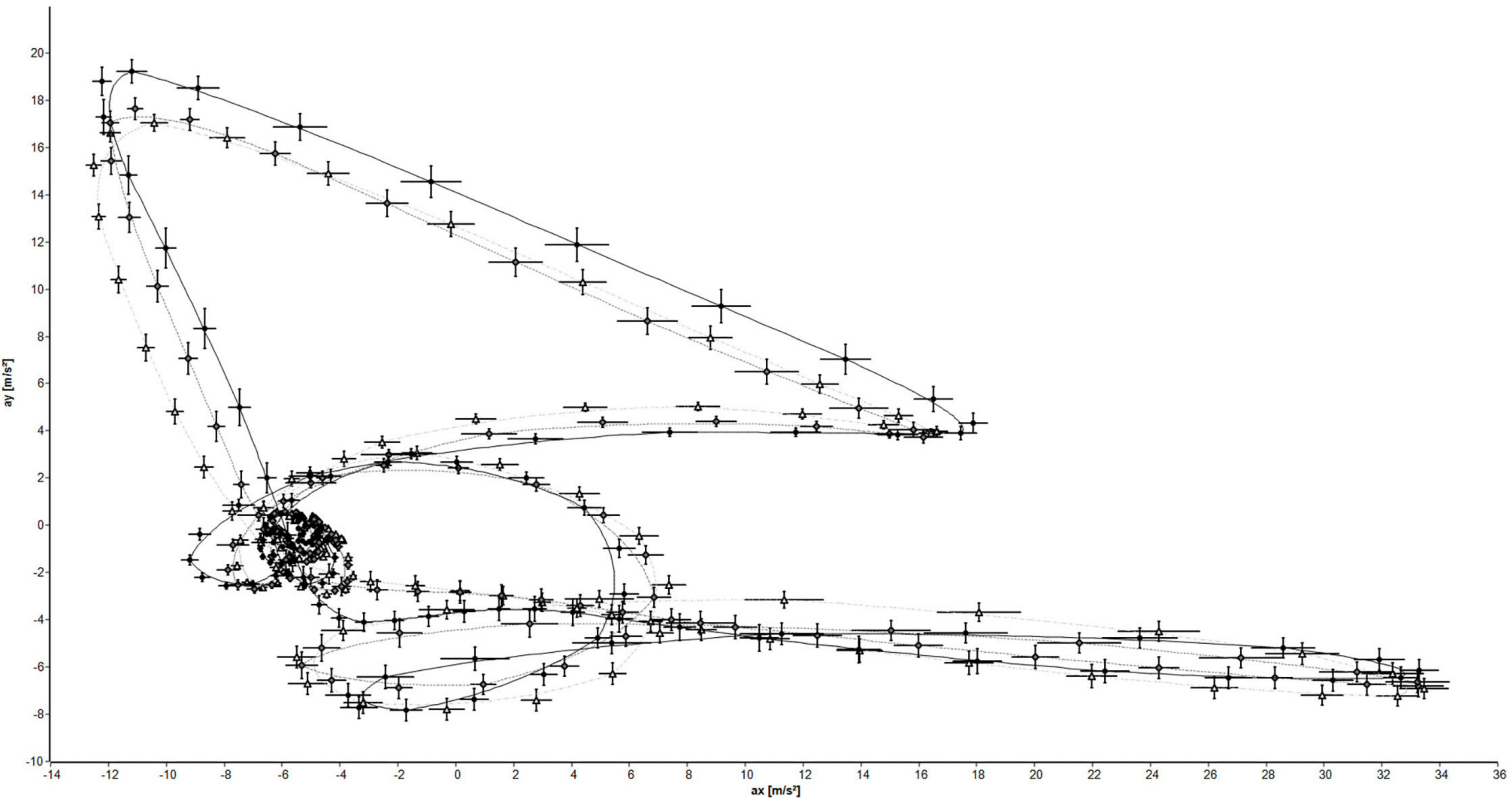
Limit-cycle attractors and standard deviation of the left leg of one person (black circles: 1 min; gray diamonds: 2 min; white triangles: 3 min).

In Figure 1, the limit-cycle attractors and the respective standard deviation of the min 1–3 of the left leg of one person are illustrated. In this representative example, it becomes visible that the limit-cycle attractor of the first minute is clearly different from those of the second and third minutes.

### Motor Performance Fatigability – Spatio-Temporal Gait Parameters

Four pwMS were categorized as having motor PF by the DWI_1−6_ (a decline from min 1–6) and only one person with MS by the DWI_2−6_ (a decline from min 2–6; Table 1).

For gait velocity, a significant main effect of time was observed (η_p_^2^ = 0.07, *F*_1.859, 76.222_ = 3.263, *p* = 0.047; Table 5). A trend was also found for stride and stance time (η_p_^2^ = 0.06, *F*_1.411, 57.845_ = 2.692, *p* = 0.093/η_p_^2^ = 0.07, *F*_1.463, 59.994_ = 2.938, *p* = 0.076). A significant time × group interaction was demonstrated for the MTC (η_p_^2^ = 0.10, *F*_1.775, 72.789_ = 4.373, *p* = 0.020) and a trend toward a time × group interaction for the stride time_CV_ and gait velocity_CV_ (η_p_^2^ = 0.05, *F*_2.679, 109.854_ = 2.319, *p* = 0.086/η_p_^2^ = 0.05, *F*_2.867, 117.531_ = 2.271, *p* = 0.087). Moreover, a main effect group could be observed for all spatio-temporal gait parameters over the 6MWT (*p* ≤ 0.05; η_p_^2^ = 0.12–0.62).

**Table 5:** Spatio-temporal gait parameters (mean ± SD) for each minute of the 6-min walk test and repeated measures ANOVAs (*p*-values and partial eta^2^ effect size).

**Gait parameter**	**Group**	**Performance per minute**						***p*** **-values**			**Partial eta^2^**		
										**Group × **			**Group × **
		**Min 1**	**Min 2**	**Min 3**	**Min 4**	**Min 5**	**Min 6**	**Time**	**Group**	**time**	**Time**	**Group**	**time**
MTC (cm)	pwMS	2.68 ± 0.63	2.58 ± 0.61	2.57 ± 0.62	2.52 ± 0.61	2.50 ± 0.61	2.84 ± 0.68	0.132	**0.021**	**0.020**	0.05	**0.12**	**0.10**
	HC	3.05 ± 0.39	3.15 ± 0.58	3.03 ± 0.58	2.96 ± 0.56	2.90 ± 0.53	2.84 ± 0.53						
MTC_CV_ (%)	pwMS	33.29 ± 14.71	35.80 ± 19.77	39.60 ± 22.68	36.95 ± 23.31	37.04 ± 18.96	30.30 ± 11.13	0.451	**0.006**	0.172	0.02	**0.17**	0.04
	HC	27.07 ± 7.37	24.45 ± 6.33	24.99 ± 5.30	26.15 ± 5.64	26.71 ± 4.99	26.68 ± 5.87						
Stride length (m)	pwMS	1.43 ± 0.16	1.41 ± 0.16	1.41 ± 0.16	1.40 ± 0.15	1.41 ± 0.14	1.40 ± 0.14	0.302	**<0.001**	0.893	0.03	**0.51**	0.00
	HC	1.67 ± 0.11	1.66 ± 0.13	1.66 ± 0.12	1.65 ± 0.12	1.66 ± 0.12	1.66 ± 0.12						
Stride length_CV_ (%)	pwMS	5.58 ± 1.92	5.56 ± 1.54	5.45 ± 2.15	6.47 ± 3.67	5.71 ± 3.06	5.83 ± 2.58	0.360	**0.001**	0.131	0.03	**0.26**	0.05
	HC	4.95 ± 2.77	3.74 ± 1.26	3.84 ± 1.43	3.72 ± 1.33	3.77 ± 1.46	3.81 ± 1.52						
Stance time (s)	pwMS	0.56 ± 0.05	0.57 ± 0.05	0.57 ± 0.05	0.57 ± 0.05	0.56 ± 0.05	0.56 ± 0.05	0.076	**<0.001**	0.263	**0.07**	**0.50**	0.03
	HC	0.48 ± 0.03	0.49 ± 0.03	0.49 ± 0.03	0.49 ± 0.03	0.49 ± 0.03	0.49 ± 0.03						
Stance time_CV_ (%)	pwMS	4.97 ± 3.81	5.49 ± 4.61	5.57 ± 4.46	6.36 ± 5.20	5.36 ± 4.33	6.00 ± 4.80	0.503	**0.003**	0.106	0.02	**0.20**	0.05
	HC	3.42 ± 1.07	2.81 ± 0.55	3.01 ± 1.15	2.68 ± 0.70	2.64 ± 0.64	2.97 ± 1.09						
Swing time (s)	pwMS	0.46 ± 0.03	0.47 ± 0.03	0.47 ± 0.04	0.47 ± 0.04	0.47 ± 0.04	0.47 ± 0.05	0.219	**0.003**	0.452	0.04	**0.20**	0.02
	HC	0.44 ± 0.02	0.44 ± 0.02	0.44 ± 0.02	0.44 ± 0.02	0.44 ± 0.02	0.44 ± 0.02						
Swing time_CV_ (%)	pwMS	5.52 ± 5.11	6.86 ± 8.06	6.99 ± 10.70	10.32 ± 13.71	8.76 ± 12.33	8.76 ± 13.29	0.321	**0.008**	0.225	0.03	**0.16**	0.04
	HC	3.21 ± 0.84	2.89 ± 0.58	2.92 ± 0.64	2.84 ± 0.62	2.78 ± 0.57	3.01 ± 0.89						
Stride time (s)	pwMS	1.02 ± 0.07	1.03 ± 0.07	1.04 ± 0.08	1.04 ± 0.08	1.02 ± 0.08	1.03 ± 0.09	0.093	**<0.001**	0.488	**0.06**	**0.42**	0.02
	HC	0.92 ± 0.04	0.93 ± 0.05	0.93 ± 0.05	0.93 ± 0.05	0.93 ± 0.05	0.93 ± 0.05						
Stride time_CV_ (%)	pwMS	2.59 ± 1.74	3.35 ± 3.05	3.55 ± 4.43	4.87 ± 5.79	4.13 ± 4.95	4.53 ± 6.27	0.340	**0.010**	0.086	0.03	**0.15**	**0.05**
	HC	2.22 ± 0.68	1.73 ± 0.37	1.86 ± 0.86	1.64 ± 0.51	1.61 ± 0.39	1.91 ± 0.82						
Velocity (m/s)	pwMS	1.41 ± 0.19	1.38 ± 0.20	1.36 ± 0.20	1.36 ± 0.19	1.38 ± 0.19	1.37 ± 0.20	**0.047**	**<0.001**	0.796	**0.07**	**0.62**	0.01
	HC	1.82 ± 0.13	1.79 ± 0.15	1.78 ± 0.15	1.78 ± 0.15	1.78 ± 0.16	1.79 ± 0.16						
Velocity_CV_ (%)	pwMS	6.42 ± 1.95	6.66 ± 2.32	6.49 ± 2.75	6.89 ± 2.71	6.43 ± 2.92	6.89 ± 3.02	0.325	**<0.001**	0.087	0.03	**0.26**	**0.05**
	HC	5.69 ± 2.97	4.24 ± 1.41	4.46 ± 1.86	4.14 ± 1.57	4.22 ± 1.64	4.43 ± 1.80						

*MTC, minimum toe clearance; HC, healthy controls; pwMS, people with Multiple Sclerosis; CV, coefficient of variation; bold, p ≤ 0.05*.

The Bonferroni *post-hoc* tests (Table 3) within each group displayed that the stance time in the first minute differed significantly from the second in HC (*p* = 0.016, d = 0.7). Additionally, a significant difference was found between the second and third and the fourth and fifth min for the MTC in HC (*p* ≤ 0.003, d = 0.8–1.1).

The *post-hoc* between-groups comparison revealed that pwMS and HC differed in all spatio-temporal gait parameters (mean and CV) from min 2 to 5 of the 6MWT (*p* ≤ 0.05, g = 0.7–2.5) significantly (Table 4). In the first minute, the groups differed only in the mean values (*p* ≤ 0.05, g = 0.7–2.6) and swing time_CV_ significantly (*p* = 0.035, g = 0.6).

**Figure 2:**
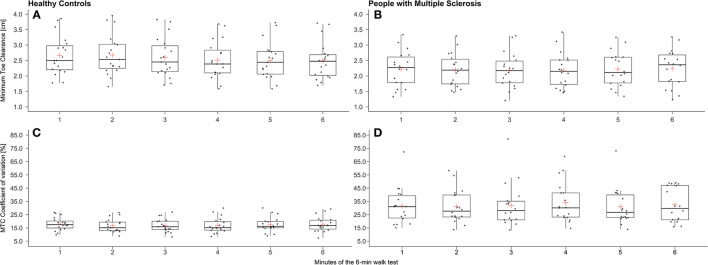
Box plots for minimum toe clearance (MTC) (**A**: healthy controls/**B**: people with Multiple Sclerosis) and its coefficient of variation (**C**: healthy controls/**D**: people with Multiple Sclerosis) of each minute of the 6-min walk test (red cross: mean).

Figure 2 illustrates the MTC and MTC_CV_ for each minute of the 6MWT. It is particularly prominent that in pwMS, the MTC_CV_ was decreased from min 5–6. Ten pwMS exhibited a decrease in the MTC_CV_ of 22.57 ± 21.41% and nine pwMS an increase of 13.46 ± 12.81% from min 5–6 (Figure 2B). Statistically, no effect could be found for these subgroups. Of these ten pwMS with decreasing MTC variability, only one exhibited motor PF detected by the DWI (−17%). The other nine pwMS had a DWI between −8 and 8%.

### Perceived Fatigability

A time effect was displayed for RPE (η_p_^2^ = 0.264, *F*_1.000, 34.000_ = 12.224, *p* = 0.001) but no time × group interaction was found. The within-group *post-hoc* tests revealed that the RPE was significantly increased in both groups from pre to post (pwMS: *p* = 0.036, d = 0.5/HC: *p* = 0.009, d = 0.6). The RPE of pwMS and HC differed significantly at both measurement time points (pre: *p* = 0.039, g = 0.7/post: *p* = 0.022, g = 0.8).

## Discussion

The main findings are that (i) gait cycles were less stable in the first compared to the second minute of the 6MWT and (ii) spatio-temporal gait parameters, including the MTC, did not change significantly over time during the 6MWT indicating no gait-related motor PF in pwMS and HC.

Regarding the first research question, we were able to demonstrate a time effect for the attractor-based gait parameters δM, δD, and D. If a system is stable, it can be expected that neighboring attractors and their variability should differ equally. The *post-hoc* tests revealed that the differences between the limit-cycle attractors (δM_2vs1min_) and between their variability (δD_2vs1min_) among the first 2 min were significantly greater than among min 2 and 3 (δM_2vs3min_/δD_2vs3min_) in pwMS. Additionally, a trend toward a time effect could be detected for the stance time in HC. Here, the *post-hoc* test showed that the first minute differed significantly from the second. Overall, these results indicate that gait performance was less stable and variability was greater in the first when compared to the second minute. This might be due to both the gait initiation process and the initial oscillations of dynamic systems at the onset of cyclic movements (transient effect) (17). Until today, the transient effect has only been proven in the context of human locomotion for running in athletes but not for walking. The transient effect during running lasted on average 5 min until the movement pattern became stable (30). However, further studies with longer walking protocols are needed to determine how long the transient effect lasts in healthy subjects and pwMS.

With regard to the second research question, *post-hoc* comparisons indicated that no deterioration of the spatio-temporal gait parameters and thus no gait-related motor PF could be detected in pwMS and HC during the 6MWT. Considering the gait velocity more closely as a commonly used measure of gait-related motor PF, both groups exhibited a U-shape over the 6MWT with the fastest velocity in the first and a similar velocity in the last minute. This pacing behavior was also found in other studies during the 6MWT in pwMS (31–33). Schwid et al. additionally reported that the pacing behavior of pwMS and HC were comparable during the 6MWT (34). In summary, these findings are in line with the results of Shema-Shiratzky et al. who showed that gait velocity over the 6MWT is not an adequate measure to quantify gait-related motor PF in pwMS (9). This applies in particular for mildly affected pwMS, as Escudero-Uribe et al. and Burschka et al. have demonstrated (35, 36). Additionally, Piérard et al. revealed that gait-related motor PF in mildly affected pwMS (EDSS 0–3) manifested an increase of the step width variability and in moderately to severely affected pwMS (EDSS ≥ 3.5) as a deterioration in walking velocity over the 500-m walk test (37). On average, the pwMS in our study were mildly affected. This might explain why no decrease in walking velocity over the 6MWT was found in the present study.

However, the results of Shema-Shiratzky et al. suggest that cadence, stride time variability, stride, and step regularity, as well as gait complexity, might be better parameters to quantify gait-related motor PF during the 6MWT. In our study, a time × group interaction could be revealed for the stride time_CV_, but the *post-hoc* tests did not indicate a significant change over time in pwMS and HC. These divergent results could be due to the fact that Shema-Shiratzky et al. compared mildly and moderately affected pwMS without including a control group and that the observed motor PF was mostly present in the moderately affected pwMS during the 6MWT.

Focusing on the MTC, a time × group interaction was found for the mean, but the *post-hoc* test did not reveal significant results regarding motor PF in pwMS. Nevertheless, the MTC_CV_ indicated a noticeable decrease from the fifth to the sixth minute in some of the pwMS. According to Nagano et al. this can be interpreted as an indicator for gait-related motor PF in the elderly (13). A similar result was also revealed by Arpan et al. (38). In this study, the authors examined gait stability over the 6MWT in pwMS and they observed that after the third minute, 60% of pwMS showed an increasingly unstable gait pattern and interpreted this as motor PF. Since no significant differences were found in the present study, it is necessary to investigate the change in MTC variability during longer and/or more intensive walking protocols to further verify this observation.

The slight increase in RPE from pre- to post-6MWT indicates that the walking protocol induced perceived fatigability in both groups with no differences between pwMS and HC. This is in line with the findings of Savci et al. who have also shown that perceived fatigue was increased slightly due to the 6MWT in both groups (39). Therefore, it seems that the walking protocol was not able to induce perceived fatigability differently in pwMS and HC. However, there are only very few studies that have examined this aspect.

Overall, the results of this study indicate that the 6MWT might be insufficient in intensity and/or duration to induce gait-related motor PF in mildly affected pwMS. This might be due to the fact that exercise intensity during the 6MWT was not sufficient to induce motor PF in our subjects. An inherent problem of walking protocols for the assessment of motor PF is that exercise intensity cannot be determined and standardized in relation to the maximal performance. This is in contrast, for example, to fatiguing cycling protocols, which define their exercise intensity as a percentage of the maximal performance achieved during an incremental performance test (e.g., percentage of peak power) (40). This approach ensures that a sufficient exercise intensity can be individually set in a standardized manner to induce motor PF. Furthermore, it enables that outcome data can be compared between individuals or groups. However, the deceleration index takes this partly into account. During this test, the maximal walking velocity over a distance of 25 feet with a dynamic start is determined and compared to the final velocity achieved during a 500-m walk test (8).

Nevertheless, it should be investigated if more intense walking protocols are suitable to induce and monitor gait-related motor PF and perceived fatigability in pwMS. For that purpose, treadmill walking protocols with increasing slope or incremental shuttle walking tests could be used, as it was done in other patient cohorts (41). However, these protocols have not yet been applied to quantify gait-related motor PF and perceived fatigability in pwMS and their feasibility needs to be verified. Besides that, there are other approaches that require longer walking protocols, such as the Fatigue Index Kliniken Schmieder, which is based on the change in gait stability and is executed over maximally 60 min or until a certain degree of perceived exhaustion (Borg RPE scale: 17) (42). However, this approach is too complex and time-consuming for everyday clinical use yet. In addition, considering our data, the calculation of the motor PF index should be revised, because the first minute is taken as a baseline for this approach (42).

Another approach to provoke a higher level of gait-related motor PF could be either to exhaust the participant cognitively beforehand (43) or to perform an additional cognitive task during walking (44–46). From these studies, it is known that both have an impact on walking performance but to the best of our knowledge, it is not known how much these interventions accelerate gait-related motor PF in pwMS.

Finally, a limitation of this study is that the sample of pwMS was on average mildly affected so that the effect of different degrees of disability on indices of gait-related motor PF and perceived fatigability could not be investigated. In future studies, mildly and moderately affected pwMS should be examined separately, because the degree of disability is an important factor for the extent of motor PF (8, 36, 47).

Another limitation is that the algorithms for the calculation of gait parameters were not validated for pwMS so far. Due to gait abnormalities often observed in pwMS, there might have been some errors in the step detection of the algorithm. Nevertheless, the degree of walking impairment was relatively low in our cohort and has probably not altered the results of the present study.

## Conclusion

In summary, it could be shown that (i) gait parameters were more stable in the second minute of the 6MWT than in the first minute in pwMS and HC (indicated by the attractor method and spatio-temporal gait parameters, respectively). In addition, (ii) no gait-related motor PF could be detected based on spatio-temporal gait parameters, including the MTC and its variability, during the 6MWT in mildly affected pwMS.

For future studies, the walking protocols should be adapted in intensity and/or duration depending on the level of disability to further investigate the transient effect but also the change in spatio-temporal gait parameters, especially in the MTC and its variability, over time. Additionally, gait parameters recorded during the first minute should be avoided as a baseline for the quantification of gait-related motor PF. Either the effect of a dynamic start has to be investigated or the gait parameters recorded during the second minute should be taken as a baseline for the assessment of gait-related motor PF in pwMS.

## Data Availability Statement

The data presented in this article are not readily available due to privacy/ethical restrictions. Requests to access the data should be directed to the corresponding author.

## Ethics Statement

The studies involving human participants were reviewed and approved by Ethics Committee of the Medical Faculty of the Otto von Guericke University (OvGU) Magdeburg (Germany). The patients/participants provided their written informed consent to participate in this study.

## Author Contributions

K-CB, LS, and CD conceptualized the study and contributed to methodology. K-CB, PB-E, and AP contributed to formal analysis and investigation. K-CB, MB, LS, AP, CD, and MJ contributed to the interpretation of data. K-CB and MB wrote the original draft. K-CB, MB, LS, CD, PB-E, and MJ contributed to writing, reviewing, and editing the manuscript. LS, CD, and MJ contributed to resources and supervision. All authors contributed to the article and approved the submitted version.

## Conflict of Interest

The authors declare that the research was conducted in the absence of any commercial or financial relationships that could be construed as a potential conflict of interest.

## Publisher's Note

All claims expressed in this article are solely those of the authors and do not necessarily represent those of their affiliated organizations, or those of the publisher, the editors and the reviewers. Any product that may be evaluated in this article, or claim that may be made by its manufacturer, is not guaranteed or endorsed by the publisher.
